# Virus replicon particle based Chikungunya virus neutralization assay using Gaussia luciferase as readout

**DOI:** 10.1186/1743-422X-10-235

**Published:** 2013-07-15

**Authors:** Sabine Gläsker, Aleksei Lulla, Valeria Lulla, Therese Couderc, Jan Felix Drexler, Peter Liljeström, Marc Lecuit, Christian Drosten, Andres Merits, Beate Mareike Kümmerer

**Affiliations:** 1 Institute of Virology, University of Bonn Medical Centre, Bonn, Germany; 2 Institute of Technology, University of Tartu, Tartu, Estonia; 3 Institut Pasteur, Biology of Infection Unit, Paris, France; 4 Inserm U1117, Paris, France; 5 Department of Microbiology, Tumor and Cell Biology, Karolinska Institutet, Stockholm, Sweden; 6 Sorbonne Paris Cité, Institut Imagine, Paris Descartes University, Paris, France

**Keywords:** Chikungunya virus, Virus replicon particles, Neutralization assay, Gaussia luciferase

## Abstract

**Background:**

Chikungunya virus (CHIKV) has been responsible for large epidemic outbreaks causing fever, headache, rash and severe arthralgia. So far, no specific treatment or vaccine is available. As nucleic acid amplification can only be used during the viremic phase of the disease, serological tests like neutralization assays are necessary for CHIKV diagnosis and for determination of the immune status of a patient. Furthermore, neutralization assays represent a useful tool to validate the efficacy of potential vaccines. As CHIKV is a BSL3 agent, neutralization assays with infectious virus need to be performed under BSL3 conditions. Our aim was to develop a neutralization assay based on non-infectious virus replicon particles (VRPs).

**Methods:**

VRPs were produced by cotransfecting baby hamster kidney-21 cells with a CHIKV replicon expressing Gaussia luciferase (Gluc) and two helper RNAs expressing the CHIKV capsid protein or the remaining structural proteins, respectively. The resulting single round infectious particles were used in CHIKV neutralization assays using secreted Gluc as readout.

**Results:**

Upon cotransfection of a CHIKV replicon expressing Gluc and the helper RNAs VRPs could be produced efficiently under optimized conditions at 32°C. Infection with VRPs could be measured via Gluc secreted into the supernatant. The successful use of VRPs in CHIKV neutralization assays was demonstrated using a CHIKV neutralizing monoclonal antibody or sera from CHIKV infected patients. Comparison of VRP based neutralization assays in 24- versus 96-well format using different amounts of VRPs revealed that in the 96-well format a high multiplicity of infection is favored, while in the 24-well format reliable results are also obtained using lower infection rates. Comparison of different readout times revealed that evaluation of the neutralization assay is already possible at the same day of infection.

**Conclusions:**

A VRP based CHIKV neutralization assay using Gluc as readout represents a fast and useful method to determine CHIKV neutralizing antibodies without the need of using infectious CHIKV.

## Background

Chikungunya virus (CHIKV) is an arthropod-borne, enveloped virus of the genus Alphavirus, family *Togaviridae*[1]. The single-stranded RNA genome of positive polarity is capped at the 5’ end and polyadenylated at the 3’ end. It is 12 kilobases (kb) in length and contains two open reading frames. The 5’ two-thirds of the genome are directly translated into the nonstructural proteins (nsP1-4) whereas the structural proteins (capsid, E1 and E2) and two small cleavage products (E3 and 6k) are translated from a subgenomic RNA, which corresponds to the last third of the genome [1]. Due to the fact that alphaviruses exhibit a broad host range (including avian, insect and mammalian cells), high levels of protein expression and a small genome that is easy to manipulate, they have been exploited as vectors for foreign gene expression or vaccination [2]. This is especially the case for Sindbis virus (SINV), Semliki Forest virus (SFV), and Venezuelan equine encephalitis virus (VEEV) [3-5]. In this context, production of virus replicon particles (VRPs) has been described, which allows the expression of foreign genes via so called single round infectious particles. Besides VRP systems, which are composed of an alphavirus replicon and one helper RNA that encodes all structural genes, VRP systems have been established in which the capsid and envelope helper regions are encoded by two separate helper RNAs [4,6,7]. This reduces the likelihood of recombination and renders the production of replication-proficient viruses (RPVs) negligible, thereby increasing biosafety [6,7].

CHIKV was first described in 1953 during an outbreak in Tanzania, East Africa [8,9]. It came into focus again due to large-scale epidemics in the Indian Ocean region starting 2005/2006 [10,11]. Since then further recurring epidemics have been observed from time to time in Africa, Indian Ocean Islands, and many parts of South-East Asia and virus emergence also occurred in European countries, like Italy and France [12-16]. CHIKV is transmitted to humans by culicids of the genus *Aedes* including *Aedes aegypti* and *Aedes albopictus*[17-19]. Infection with the virus results in Chikungunya fever (CHIKF), which is characterized by high fever, fatigue, headache, nausea, vomiting, rash, myalgia and severe arthralgia [8,20,21]. To date, there is no specific treatment and no licensed vaccine available against infection with CHIKV.

To determine the immune status of a patient, a neutralization (NT) assay is necessary. Furthermore, NT assays represent an important method to validate the efficiency of potential vaccines during vaccine development. Most neutralization assays described so far for CHIKV involve the use of infectious CHIKV particles. Besides plaque reduction neutralization assay, NT assays using immunofluorescence or inhibition of the cytopathogenic effect (CPE) as readouts have been described [22-26]. Recently, a CHIKV pseudotyped lentiviral vector-based NT assay was established [27]. The latter circumvents the use of infectious CHIKV particles and readout was performed several days after transduction by measuring luciferase activity in the cells transduced with the CHIKV-pseudotyped lentiviral vector [27]. The aim of our studies was to also develop an NT assay for CHIKV infection, which is independent of infectious particles but in addition allows a very fast and easy readout. Several reporter proteins have been described, which allow to follow up viral infection or replication. Besides fluorescent proteins reporter proteins based on bioluminescent reactions are widely used. The latter include for example Renilla (R) or Firefly (F) luciferase (luc) but also Gaussia luciferase (Gluc), which in contrast to Rluc and Fluc is secreted into the supernatant thereby facilitating analyses [28,29]. Hence, we aimed to establish a CHIKV VRP based NT assay using the oxidative decarboxylation of coelenterazine catalyzed by secreted Gluc leading to emission of blue light as readout.

## Results

### Establishment of CHIKV replicon and helper constructs

For the production of CHIKV replicon particles a split helper system should be applied since it has been described that providing the structural proteins via two separate helper constructs did not result in detectable incidental production of infectious particles due to recombination [6,7].

**Figure 1:**
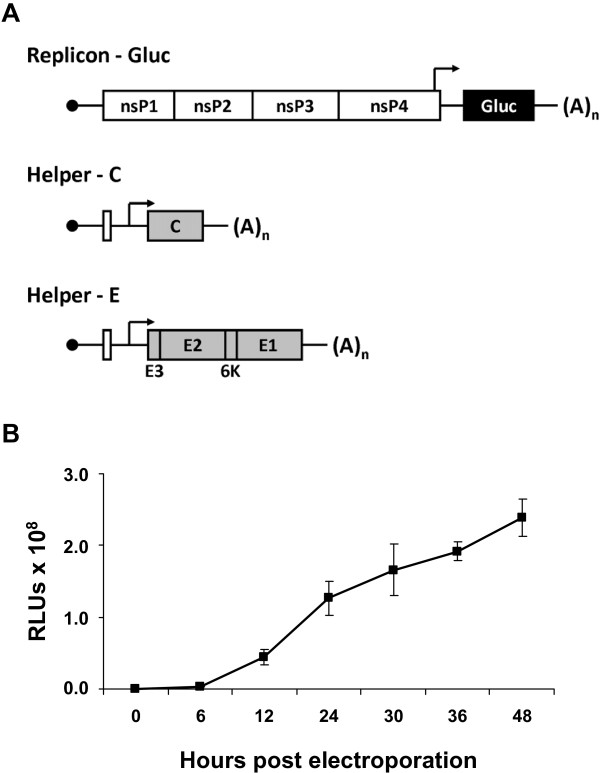
**CHIKV VRP system.** (**A**) Schematic presentation of the CHIKV replicon expressing Gluc marker and the CHIKV helper-C and helper-E RNAs. Lines represent non-translated regions and boxes represent translated regions, whereas white boxes indicate nonstructural proteins and gray boxes indicate structural proteins. The Gluc reporter is represented as black box. In the helper constructs 5’ terminal nucleotides of the nsP1 gene important for RNA replication were retained. Arrows indicate the position of the subgenomic promoter. The solid black circle at the 5’ end of each RNA represents the CAP structure; (A)_n_ indicates the poly(A) tail. (**B**) Kinetics of Gluc secretion after electroporation of CHIKV replicon expressing Gluc. *In vitro*-synthesized replicon RNA was electroporated into BHK cells and release of Gluc into the supernatant was measured at the indicated time points.

A CHIKV replicon was established, in which the structural genes were replaced by a reporter gene (Figure 1A). For easy readout, Gluc was chosen as reporter protein, since it is secreted into the supernatant and therefore allows easy readout without the need of preparing cell lysates. Furthermore, two helper plasmids were established expressing either the CHIKV capsid protein C (pChikHelper-C) or the CHIKV envelope proteins p62-6K-E1 (pChikHelper-E). The helper RNAs contain the 5’ and 3’ CHIKV replication signals and a subgenomic promoter followed by either the capsid gene or the remaining structural proteins, respectively (Figure 1A). The helper RNAs as well as the CHIKV replicon RNA can be produced *in vitro* from plasmids, which contain these sequences under control of an SP6 promoter.

To prove efficient secretion of Gluc from the replicon *in vitro* transcribed replicon RNA was electroporated into baby hamster kidney-21 (BHK-21) cells. After electroporation, increasing amounts of Gluc could be detected over time in the supernatant demonstrating the efficient replication and secretion of Gluc of the established CHIKV replicon (Figure 1B).

### Conditions for optimized VRP production

**Figure 2:**
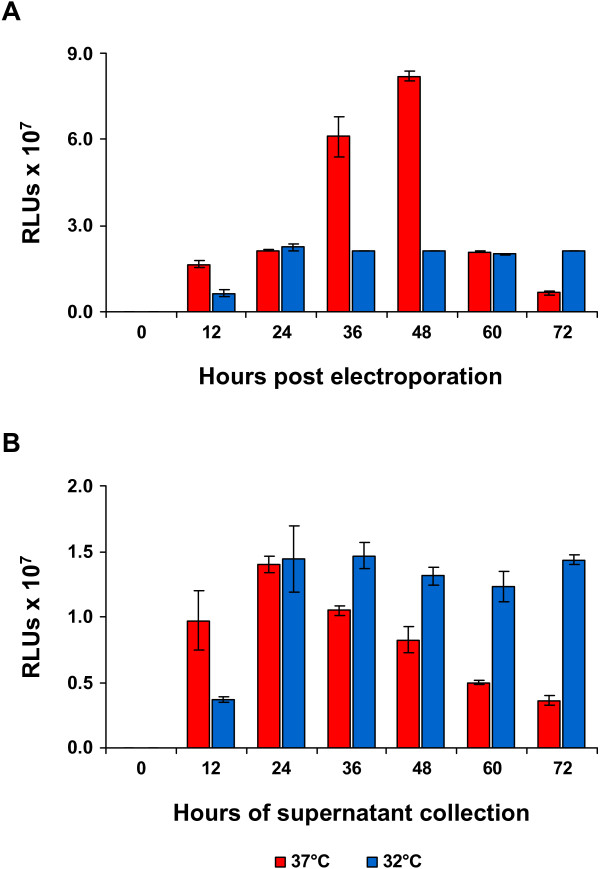
**Optimum of VRP production.** (**A**) *In vitro*-synthesized Gluc replicon RNA was coelectroporated with *in vitro*-synthesized helper-C and helper-E RNA and cells were either incubated at 32°C or 37°C. Supernatants were harvested at the indicated time points after electroporation and Gluc activity was measured in RLUs. (**B**) Same volumes of supernatants harvested at different time points from the different incubation temperatures in (**A**) were used to infect fresh BHK cells at 37°C. At 6 h post infection, Gluc activity was measured in the supernatant.

For production of VRPs the CHIKV Gluc replicon was coelectroporated with the two helper RNAs into BHK-21 cells. It has been observed that VRP production is more efficient at temperatures lower than 37°C. Hence, to compare VRP production at different temperatures, coelectroporated cells were incubated at either 37°C or 32°C and the activity of secreted Gluc was measured in Relative Light Units (RLUs). Whereas at 37°C Gluc secretion increased over time until 48 h post electroporation before dropping down again, Gluc levels remained lower and more constant at lower temperature (Figure 2A). Interestingly, when the supernatants were passaged once on BHK cells to analyze for produced VRPs it turned out that VRPs had been released to higher and more constant levels for the experiment performed at 32°C (Figure 2B). This suggests that although Gluc was more efficiently secreted at 37°C the VRP production was indeed more stable at lower temperature. Hence, for optimized VRP production, the coelectroporated cells were incubated at 32°C and VRPs were harvested at 36 h post electroporation. Harvested VRPs were purified via a sucrose cushion. As determined by CHIKV real-time PCR, the 30-fold concentrated VRP stocks contained around 1 × 10^9^ viral RNA copies/ml.

### Comparison of test conditions with regard to VRP infection rate, plate format and readout time

**Figure 3:**
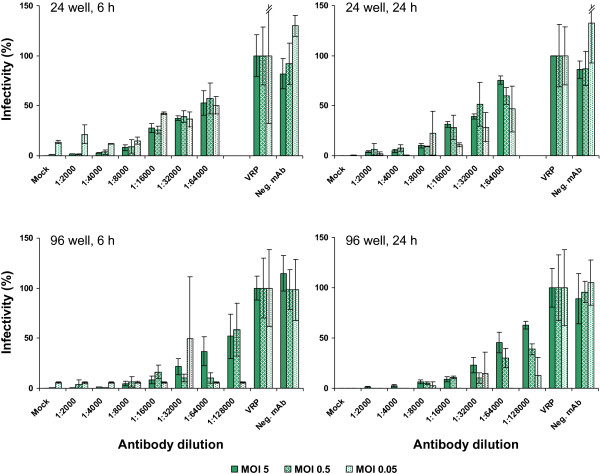
**VRP based NT assay using a monoclonal antibody.** Assays were performed in either 24- or 96-well format as indicated. Neutralizing monoclonal antibody directed against CHIKV E2 protein was serially diluted and preincubated with VRPs corresponding to an MOI of 5, 0.5 or 0.05, respectively. A monoclonal antibody against CHIKV capsid protein was used as control (Neg. mAb). Preincubated samples were used to infect the BHK cells in the 24- or 96-well plates and readout via Gluc secreted into the supernatant was performed at 6 or 24 h post infection as indicated. The bar labeled with VRP represents infection with the appropriate amount of VRPs not preincubated with monoclonal antibody. The bar labeled with Mock represents the background measured in untreated (non-infected) BHK cells. The % infectivity was normalized to VRP infection without monoclonal antibody incubation. Data represent means and standard deviation of experiments performed in triplicate.

For comparable analyses same test conditions need to be applied. These include among others parameters like the amount of VRPs used in the NT assay as well as the readout time or the plate format used. Even though the VRP concentration in established stocks can be determined via real-time PCR to calculate defined infection rates it might be desirable to use the same VRP batch for as many NT assays as possible. NT assays using different amounts of VRPs corresponding to multiplicities of infection (MOIs) of 5, 0.5 and 0.05 calculated based on the determined RNA copies in the VRP stock were evaluated. Another reason for testing also lower MOIs was that this better reflects the conditions used in classical plaque neutralization assays. There, a low MOI needs to be chosen to be able to count single plaques for readout. Evaluation of different MOIs was first carried out using the neutralizing monoclonal antibody (mAb) D3.62 directed against CHIKV E2 (T. Couderc and M. Lecuit, unpublished data). The respective experiments were performed in either a 24- or 96-well format and readout was done at 6 and 24 h post VRP infection (Figure 3). To evaluate the neutralization activity, the infectivity rates measured via Gluc secretion retained after VRP incubation with the respective antibody dilution were determined in percent compared to the VRP only control.

As shown in Figure 3, stepwise neutralization was observed after applying the monoclonal antibody in different dilutions. In the 24-well format the most consistent data were obtained when VRPs were used at MOI 5 or MOI 0.5. Decreasing the MOI to 0.05 increased the deviation from the mean. For the 96-well plate format MOI 5 yielded the most reliable data whereas the number of outliers increased at lower MOI. The least reliable data were obtained in the 96-well format using an MOI of 0.05. Comparing the data received after evaluating the NT assay at 6 h post infection (p.i.) versus 24 h p.i. revealed similar results indicating that the readout of the Gluc VRP assay readily can be performed within one day.

**Figure 4:**
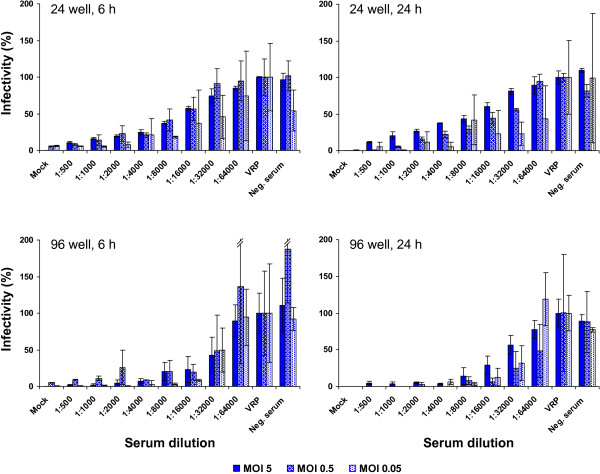
**VRP based NT assay using patient serum.** Assays were performed in either 24- or 96-well format as indicated. Human serum from a CHIKV patient was serially diluted and preincubated with VRPs corresponding to an MOI of 5, 0.5 or 0.05, respectively. Preincubated samples were used to infect the BHK cells in the 24- or 96-well plates and readout via Gluc secreted into the supernatant was performed at 6 or 24 h post infection as indicated. The bar labeled with VRP represents infection with the appropriate amount of VRPs not preincubated with patient sera. The bar labeled with Mock represents the background measured in untreated (non-infected) BHK cells. Negative (Neg.) serum from a person not infected with CHIKV was used as control. The % infectivity was normalized to VRP infection without serum incubation. Data represent means and standard deviation of experiments performed in triplicate.

**Figure 5:**
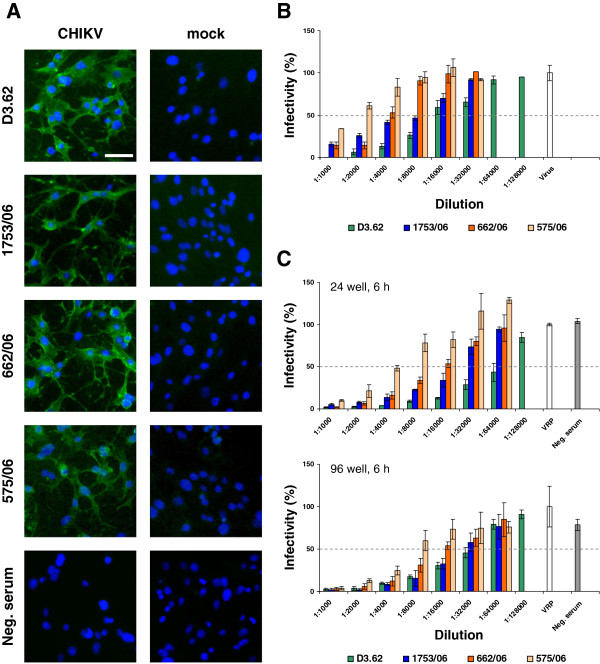
**Comparison of plaque neutralization and VRP based NT assays.** (**A**) CHIKV infected and non-infected (mock) BHK cells were used for indirect immunofluorescence analyses using the indicated monoclonal antibody or human sera. Nuclei were stained with DAPI. The bar represents 25 μm. (**B**) For the plaque neutralization assay, serial dilutions of monoclonal antibody D3.62 or three patient sera were preincubated with infectious CHIKV. NT assays were performed in 6-well format with readout at 48 h p.i. using crystal violet staining. The bar labeled with Virus represents a non-neutralized infectivity control, which was set 100%. The % infectivity was normalized to the non-neutralized virus infection. The gray dotted line indicates the 50% infectivity threshold. Data represent the means and ranges of duplicate infection experiments. (**C**) For the VRP based NT assay, serial dilutions of monoclonal antibody D3.62 or three patient sera were preincubated with VRPs. NT assays were performed in either 24-well plates (top panel) or 96-well plates (bottom panel) using VRPs at an MOI of 5. Readout was performed at 6 h p.i. via measurement of Gluc secreted into the supernatant. The bar labeled with VRP represents infection with the appropriate amount of VRPs not preincubated with patient sera/antibody. Negative (Neg.) serum from a person not infected with CHIKV was used as control. The % infectivity was normalized to VRP infection without serum/antibody incubation. The gray dotted line indicates the 50% infectivity threshold. Data represent average and standard deviation of experiments performed in triplicate.

Similar results were obtained when the same set of conditions was tested using a human serum from a CHIKV patient (1753/06) (Figure 4). Using immunofluorescence analysis, the serum was confirmed to be positive against CHIKV (see also Figure 5A).

Whereas the human CHIKV patient serum resulted in dose dependent neutralization, a negative serum from a healthy person did not show any neutralization activity (Figure 4). Again, MOI 5 and MOI 0.5 turned out to be the most applicable conditions in the 24-well format and MOI 5 yielded the most consistent results in the 96-well format (Figure 4).

### Evaluation of VRP based neutralization assay in comparison to plaque neutralization assay

To further evaluate the applicability of the established VRP based NT assay comparison to a classical plaque neutralization assay based on infectious CHIKV was drawn. The respective analysis was done using again the neutralizing monoclonal antibody D3.62, the patient serum 1753/06, as well as two further sera (662/06 and 575/06) from CHIKV patients diseased during the 2006 epidemic in the Indian Ocean region [30]. All sera as well as the monoclonal antibody were confirmed to be reactive against CHIKV using indirect immunofluorescence (Figure 5A). The plaque neutralization assay was performed in a 6-well plate format using 100 plaque forming units (PFU) per well (Figure 5B), whereas for the VRP based NT assay both the 24-well and 96-well format was applied using MOI 5 (Figure 5C). The neutralization activity of a serum is usually judged by determining the serum dilution, which results in 50 or 80% reduction of infectivity (NT_50_ or NT_80_ titer, respectively). When using different methods of NT assays, comparison of absolute NT_50_ values is limited due to different test conditions. However, the order of neutralizing activity among antisera tested should be the same. Table 1 provides details on the predicted NT_50_ values using the data sets depicted in Figure 5B and 5C. Overall, the NT_50_ titers determined via the classical plaque neutralization assay were lower than those observed in the VRP assays (Table 1). Nevertheless, the same order with regard to neutralizing activity of the samples was obtained for all NT assay types, with the monoclonal antibody neutralizing the best and serum 575/06 neutralizing the least (Table 1). This comparison was supported by narrow 95% confidence intervals (CI) determined for each serum (Table 1). In the 96-well VRP assay format, all 95% CI overlapped partially. In contrast, the 95% CI overlapped marginally only for two sera in the plaque assay, and no overlap at all was observed in the 24-well VRP assay format. Hence, the CHIKV VRP based NT assay represents a useful alternative to CHIKV NT assays involving infectious virus and in addition allows a fast and easy analysis of neutralizing antibodies in serum samples that can be performed within one day.

**Table 1:** **Neutralizing antibody titers (NT**_**50**_**)**

	**Virus-NT**^***a***^		**VRP-NT (24 well)**^***b***^		**VRP-NT (96 well)**^***b***^	
**mAb/ Serum**	**NT**_**50**_^***c***^	**(95% CI)**^***e***^	**NT**_**50**_^***d***^	**(95% CI)**	**NT**_**50**_^***d***^	**(95% CI)**
D3.62	**16256**	(13465 – 19731)	**58306**	(45196 – 79754)	**26885**	(22799 – 32054)
1753/06	**5978**	(4882 – 7350)	**16203**	(12911 – 20881)	**26314**	(21004 – 34401)
662/06	**3582**	(2581 – 5061)	**13437**	(11819 – 15382)	**18743**	(15050 – 24194)
575/06	**1375**	(615 – 2183)	**4511**	(3590 – 5662)	**10911**	(7743–15876)

*a* Neutralization assay performed with virus particles (plaque assay).

*b* Neutralization assay performed with virus replicon particles (Gluc assay).

*c* NT_50_ values were determined via probit analysis using the data set of Figure 5B.

*d* NT_50_ values were determined via probit analysis using the data sets of Figure 5C.

*e* 95% confidence interval.

## Discussion

The present study shows that Gluc expressing VRPs represent a useful tool to determine neutralizing antibodies without the need of infectious CHIKV particles. VRPs were produced by cotransfection of a CHIKV replicon expressing Gluc and two helper RNAs. First studies describing the production of VRPs involved the cotransfection of a single helper RNA encoding all structural proteins [3,31]. However, in this constellation a single RNA recombination event is sufficient to reproduce a full-length genome resulting in production of infectious particles [31]. Expressing the structural proteins via two helper RNAs circumvents this problem and it was shown that using this strategy production of infectious particles due to recombination is negligible [2,6], which is an important aspect with regard to biosafety issues.

For certain alphaviruses, like SFV and SINV, expression of the structural proteins was described to be dependent on an enhancer sequence located in the 5’ terminal part of the sequence encoding the capsid protein [32,33]. To ensure in these cases efficient translation of the envelope proteins in a bipartite helper packaging system, the enhancing sequence of the capsid protein was fused 5’-terminally to the envelope genes [6,7]. However, for VEEV a split helper system was described in which the envelope proteins were expressed without a capsid translation enhancer [4]. The latter implicates that the enhancer sequence is not necessarily needed in the VEEV context [4]. This seems to be also the case for CHIKV, as no stable hairpin structure comparable with capsid enhancers of SFV and SINV could be predicted in the corresponding region of the CHIKV genome. Nevertheless, it was also observed that the CHIKV replicon could be efficiently packed using split helper RNAs of SFV (A. Merits and A. Lulla, unpublished data). Therefore the effect of classical SFV capsid enhancer was tested also in the context of CHIKV helper RNAs. In this experiment the presence of enhancer sequence not only failed to increase but reduced the VRP production (A. Lulla, V. Lulla, unpublished data). Therefore in our split helper system the helper-E construct used did not contain any capsid enhancer and still allowed efficient production of VRPs.

Alphaviruses are known to be transmitted by insect vectors, which exhibit body temperatures below 37°C. Hence, the VRP production was also tested at lower temperature and has proven to be more efficient at 32°C than at 37°C. Reducing the temperature to 28°C even slightly increased the VRP yield further but simultaneously slowed down the production process resulting in a prolonged production time (data not shown).

To facilitate and accelerate the readout of the NT assay we established a VRP system expressing Gluc, which is secreted into the supernatant [28]. Hence, measuring can directly be performed from the supernatant without the need of lysing cells. The latter was described to be necessary for the pseudotyped lentiviral vector-based CHIKV NT assay, which used Fluc as reporter protein [27]. In addition, the humanized Gluc has been described to be 1000-fold more sensitive compared to humanized Rluc or Fluc [28,34]. Furthermore, Gluc is very stable (half-life around 6 days [35]), which allows storage of the supernatant at 4°C for several days without significantly loosing activity [28]. On the other hand, due to the high sensitivity, Gluc reporter assays are likely to be prone to pipetting errors [28]. Hence, pipetting smaller volumes when working in the 96-well format at lower MOI might be one reason for the higher deviations observed in this experimental setup. As already described, it is not recommended to use a pipetting volume below 10 μl for Gluc assays [28]. Nevertheless, when using an MOI of 5, reliable results were also obtained in the 96-well format. Using an even higher MOI is not recommended. Besides consuming excessive amounts of VRPs, the risk to exhaust the assay exists. Also, especially at 24 h post infection, the amount of Gluc released into the supernatant after using an MOI of 50 was so high that samples had to be diluted to allow measurement of Gluc activity (data not shown). This could result in an additional source of variation.

The 96-well format might especially be favorable to use when only small sample volumes are available or when neutralizing titers have to be determined for an extensive number of samples. Working in a 96-well format allows to directly transfer the Gluc containing supernatant from the NT assay plate to a corresponding 96-well readout plate using a multichannel pipette thereby avoiding time consuming single pipetting steps. Furthermore, both the recently described pseudotyped lentiviral vector-based NT assay [27] and our VRP based NT assay have the advantage that the use of infectious CHIKV particles is avoided. However, for the lentiviral system readout is performed several days after transduction, whereas our VRP based assay allows carrying out the NT assay including readout within one day.

Performing different types of NT assays results in fairly different scales of NT titers. This was also observed when comparing the lentiviral vector-based NT assay with a classical plaque neutralization assay [27]. Similarly, the antibody dilutions for which the infectivity was inhibited by 50% were different for each sample comparing our VRP based assay with a plaque neutralization assay. Nevertheless, the order of neutralization potency among the samples was consistent (Table 1) indicating that the assay is suitable to determine comparative neutralization activities. Furthermore, the fact that no infectious CHIKV particles are needed and that readout can already be performed after 6 h makes the established Gluc VRP based NT assay a valuable tool to study patient or animal serum samples. Besides diagnostic purposes, analyses of neutralizing antibodies in human sera will help to understand immune mechanisms involved in CHIKV disease and analyses in animals will be useful in evaluation steps during CHIKV vaccine development.

## Conclusions

We have established an NT assay based on CHIKV VRPs using secreted Gluc as readout. This circumvents the use of infectious CHIKV and allows an easy readout. The VRP based assay can be performed in microtiter plates and readout can be done within a single day making it suitable for high-throughput analyses of CHIKV neutralization antibodies in human or animal sera.

## Methods

### Cells and viruses

Baby hamster kidney-21 (BHK-21) cells were maintained in Glasgow´s Minimum Essential Medium (GMEM) supplemented with 5% fetal bovine serum (FBS), 1% L-glutamine, 10% tryptose phosphate broth, 20 mM HEPES pH 7.2, 100 U/ml penicillin and 0.1 mg/ml streptomycin at 37°C and 5% CO_2_.

A stock of recombinant wild-type CHIKV (CHIKV-LR2006 OPY1, [36]) was produced on BHK-21 cells. Cells were infected at an MOI of 0.1 for 1 h and virus harvested from the supernatant 2 days p.i. was stored at −80°C. Experiments with infectious CHIKV were performed in a biosafety level 3 laboratory.

### Antibodies and antisera

Monoclonal antibody D3.62 (35 mg/ml) is directed against CHIKV E2 protein (T. Couderc and M. Lecuit, unpublished data). Human sera containing neutralizing CHIKV antibodies were obtained from patients returning to Europe from the Indian Ocean region in 2006 and have been described previously [30]. Briefly, sera from patient 575/06 returned from the Seychelles, patient 662/06 from La Réunion and patient 1753/06 from Mauritius. Monoclonal antibody Z2G2 recognizing CHIKV capsid protein was kindly provided by Petra Emmerich (Bernhard Nocht Institute, Hamburg, Germany).

### Indirect immunofluorescence

BHK-21 cells were cultured on glass coverslips and infected with CHIKV-LR2006 OPY1 at an MOI of 0.5. At 24 h after infection, cells were fixed with ice-cold methanol / acetone (1:1) and air-dried. Serum samples or monoclonal antibodies were diluted 1:5000 in PBS and incubated for 1 h at 37°C on fixed cells. Serum antibodies were detected by Alexa 488-labeled goat anti-human IgG (Jackson ImmunoResearch, 1:500) and monoclonal antibodies by cyanine 3-conjugated goat anti-mouse IgG (Jackson ImmunoResearch, 1:200) using fluorescence microscopy (Axiovert 40 microscope, Zeiss). Nuclei were stained with 4',6-diamidino-2-phenylindole (DAPI).

### Plasmid constructs

The construction of full-length infectious cDNA of CHIKV-LR2006 OPY1 is described elsewhere [36]. To obtain plasmid pChikRepl containing the cDNA of a CHIKV replicon, the region of the infectious cDNA clone corresponding to the coding sequence of CHIKV structural proteins (nucleotides 7565–11310 of CHIKV-LR2006 OPY1) was replaced with sequence 5’ CCTAGGTAATAAGTTTAAAC 3’ (recognition sites of restriction endonucleases *Avr*II and *Mss*I (*Pme*I) are underlined) by PCR-mediated mutagenesis. The coding sequence of Gluc (optimized for human codon usage, synthesized by GeneArt (Life Technologies)) was PCR amplified using primers 5’ TATTCCTAGGCCACC**ATG**GGAGTCAAAGTTCTGTTTGCC 3’ (start codon is in bold, recognition site of *Avr*II restriction enzyme is underlined) and 5’ TGATGTTTAAAC**TTA**GTCACCACCGGCCCCCTTGATCTT 3’ (stop codon is in bold, recognition site of *Mss*I restriction enzyme is underlined); the obtained fragment was digested with *Avr*II and *Mss*I enzymes (Thermo Scientific, USA) and inserted into pChikRepl vector digested with the same enzymes. The resulting plasmid was designated as pChikRepl-Gluc. To obtain a plasmid, containing the cDNA of a packaging construct for ChikRepl-Gluc, the region of the infectious cDNA corresponding to nucleotides 308–7419 of CHIKV-LR2006 OPY1 [36] was replaced by sequence 5’ GTTTAAAC 3’ (recognition site of *Mss*I restriction enzyme) using PCR-mediated mutagenesis; the resulting plasmid was designated pChikHelper. To obtain plasmids for a split helper system the following changes were introduced into pChikHelper using PCR based mutagenesis. First, to obtain pChikHelper-E the region corresponding to nucleotides 461–1248 (coding region for capsid protein) of pChikHelper was replaced by sequence 5’ CCTAGGCCACCATG 3’ (recognition site of *Avr*II restriction enzyme is underlined). Second, to obtain pChikHelper-C the region corresponding to nucleotides 1246–4206 (coding region for E3-E2-E1 glycoproteins) of pChikHelper was replaced by sequence 5’ TAAGTTTAAAC 3’ (recognition site of *Mss*I restriction enzyme is underlined). All constructs were verified using Sanger sequencing. Sequences of all plasmid vectors are available from authors upon request.

### Electroporation and VRP production

Replicon and helper DNA templates were linearized with *Not*I and *in vitro* transcribed using the mMESSAGE mMACHINE SP6 Kit (Ambion). For recovery of VRPs 1 μg of each, the replicon and helper RNAs, were coelectroporated into 1 × 10^6^ BHK-21 cells using BHK-21 preset protocol of Gene Pulser Xcell (Bio-Rad). Cells were seeded into 25 cm^2^ flasks and incubated at 32°C for 36 h. For large-scale VRP production supernatants of six electroporations were pooled for purification. After removing detached cells and cell debris by clarifying for 30 min at 4000 g and 4°C, the supernatants were passed through a 0.45 μm filter and applied to a 20% sucrose cushion followed by centrifugation for 90 min at 25000 rpm and 4°C (SW 32 Ti rotor, Beckman Coulter). Pellets were resuspended in TNE buffer (50 mM Tris–HCl, pH 7.4, 100 mM NaCl, 0.5 mM EDTA) over night at 4°C before passing through a 0.22 μm filter. VRPs were stored at −80°C.

### Real-time reverse transcription-PCR

RNA from VRPs in the cell culture supernatant or after sucrose cushion purification was extracted using NucleoSpin RNA Virus Kit (Macherey-Nagel). The isolated RNA was detected by real-time reverse transcription-PCR (RT-PCR) using the SuperScript III One-Step RT-PCR System with Platinum *Taq* DNA polymerase (Invitrogen). For detection of CHIKV RNA, the 25 μl reaction contained 3 μl of RNA, 1x Reaction Mix, 0.5 μg BSA, 0.5 μl SS III RT / Platinum Taq Mix, 0.6 μM of primer CHIKSI, 0.6 μM of primer CHIKASI and 0.2 μM of the CHIKP probe [30]. Thermocycling was performed on a LightCycler 480 (Roche) programmed for: 30 min at 50°C for reverse transcription, 2 min at 94°C to activate the *Taq* polymerase and 45 PCR amplification cycles of 15 sec at 94°C, 30 sec at 58°C and 30 sec at 72°C. Photometrically quantified *in vitro*-RNA transcripts of the target regions were used in the PCR to generate a standard curve for viral RNA quantification.

### Luciferase assay

Gluc was measured from the supernatant of infected cells using *Renilla* Luciferase Assay System (Promega) according to the manufacturer’s instructions. Luciferase activity was measured in RLUs. Measurement was performed automated in a Synergy 2 microplate reader (BioTek) using polystyrol microplates (Greiner Bio-one).

### VRP neutralization assay

The day before infection, 24- or 96-well plates were seeded with 1 × 10^5^ or 2 × 10^4^ BHK-21 cells per well, respectively. VRPs were applied in the NT assay at MOI 5, 0.5 or 0.05 (calculated based on VRP RNA copies/ml). All dilutions were performed using GMEM supplemented with 1% FBS. VRP dilutions were incubated with serially diluted antibody or human serum for 1 h at 37°C before adding the mixture to a monolayer of BHK-21 cells in either a 24-well plate (total volume per well 200 μl) or a 96-well plate (total volume per well 40 μl). After incubation for 1 h at 37°C the inoculum was removed, cells were washed once with PBS and medium was added. Supernatants for Gluc measurement were taken at 6 h and 24 h p.i. Neutralization potency was determined as percentage of measured Gluc activity compared to Gluc readout after VRP application without antibody/serum.

### Plaque reduction neutralization assay

About 100 PFU of infectious wild-type virus were incubated with serially diluted antibody or human serum for 1 h at 37°C, added to a monolayer of BHK-21 cells in a 6-well plate (total volume per well 600 μl) and incubated at 37°C for 1 h. Subsequently, the inoculum was replaced by an overlay containing 0.6% agarose in MEM. At 48 h p.i. cells were fixed with 7% formaldehyde and plaques were visualized using crystal violet staining (1% crystal violet in 50% ethanol). Neutralization potency was determined as percentage of plaque titers compared to plaque titers after virus infection without antibody/serum.

### Statistics

Probit analysis for determination of NT_50_ values was done with the SPSSV21 software package (IBM, Ehningen, Germany).

## Competing interests

The authors declare that they have no competing interests.

## Authors’ contributions

SG, BMK conceived and designed experiments; AL, VL, AM designed and established constructs for VRP production; TC, ML, CD contributed CHIKV antibody/sera; JFD performed statistical analyses; PL participated in optimized VRP production; SG, AM, BMK wrote the paper. All authors have read and approved the manuscript.
